# The Effect of Hydrogen on the Stress-Strain Response in Fe_3_Al: An ab initio Molecular-Dynamics Study

**DOI:** 10.3390/ma14154155

**Published:** 2021-07-26

**Authors:** Petr Šesták, Martin Friák, Mojmír Šob

**Affiliations:** 1 Central European Institute of Technology (CEITEC), Brno University of Technology, Technická 2, CZ-616 69 Brno, Czech Republic; sestak@fme.vutbr.cz; 2 Faculty of Mechanical Engineering, Brno University of Technology, Technická 2, CZ-616 69 Brno, Czech Republic; 3 Institute of Physics of Materials, v.v.i., Czech Academy of Sciences, Žižkova 22, CZ-616 62 Brno, Czech Republic; mojmir@ipm.cz; 4 Department of Chemistry, Faculty of Science, Masaryk University, Kotlářská 2, CZ-611 37 Brno, Czech Republic

**Keywords:** Fe3Al, hydrogen, embrittlement, molecular dynamics, strength, ab initio, fracture

## Abstract

We performed a quantum-mechanical molecular-dynamics (MD) study of Fe${}_{3}$Al with and without hydrogen atoms under conditions of uniaxial deformation up to the point of fracture. Addressing a long-lasting problem of hydrogen-induced brittleness of iron-aluminides under ambient conditions, we performed our density-functional-theory (DFT) MD simulations for T = 300 K (room temperature). Our MD calculations include a series of H concentrations ranging from 0.23 to 4 at.% of H and show a clear preference of H atoms for tetrahedral-like interstitial positions within the D0${}_{3}$ lattice of Fe${}_{3}$Al. In order to shed more light on these findings, we performed a series of static lattice-simulations with the H atoms located in different interstitial sites. The H atoms in two different types of octahedral sites (coordinated by either one Al and five Fe atoms or two Al and four Fe atoms) represent energy maxima. Our structural relaxation of the H atoms in the octahedral sites lead to minimization of the energy when the H atom moved away from this interstitial site into a tetrahedral-like position with four nearest neighbors representing an energy minimum. Our ab initio MD simulations of uniaxial deformation along the 〈001〉 crystallographic direction up to the point of fracture reveal that the hydrogen atoms are located at the newly-formed surfaces of fracture planes even for the lowest computed H concentrations. The maximum strain associated with the fracture is then lower than that of H-free Fe${}_{3}$Al. We thus show that the hydrogen-related fracture initiation in Fe${}_{3}$Al in the case of an elastic type of deformation as an intrinsic property which is active even if all other plasticity mechanism are absent. The newly created fracture surfaces are partly non-planar (not atomically flat) due to thermal motion and, in particular, the H atoms creating locally different environments.

## 1. Introduction

Our theoretical study focuses on the impact of hydrogen in Fe${}_{3}$Al as a material belonging to a very promising class of iron aluminides. Some members of this family of materials possess very interesting properties including remarkable resistance to oxidation, relatively low density, electrical resistivity, or low cost of raw materials [1,2,3,4,5,6,7,8,9,10,11,12,13]. Consequently, the Fe-Al-based materials have been intensively studied both experimentally (see, e.g., Refs. [14,15,16,17,18,19,20,21,22,23,24,25,26,27,28,29,30,31,32,33,34,35,36,37]) and theoretically [38,39,40,41,42,43,44,45,46,47,48,49,50,51,52,53,54,55,56,57,58,59,60,61,62,63].

There are, unfortunately, two major problems associated with the Fe-Al-based materials that hinder their wider industrial use. One of them is their limited ductility at ambient temperatures (see, e.g., Ref. [64]) and the other one is their low strength and creep resistance at high temperatures [4]. Our study aims at shedding a new light on the former issue of room-temperature brittleness.

The lack of ductility in the case of iron-aluminides turned out to be environment-induced (see, e.g., Refs. [64,65,66,67]) and, importantly, could be partly suppressed [68,69]. As the brittleness demonstrates itself in particular in the presence of water vapor in the atmosphere surrounding the samples it is associated with hydrogen (see, e.g., an overview paper [70] with a list of earlier experimental works supporting this interpretation). As a study identifying hydrogen as the primary cause of the embrittlement, we can mention the work by Alven and co-workers [71]. Those authors analyzed tensile and fatigue crack growth behavior of several Fe–Al alloys covering a range of Al content from 16 to 35 at.% in different environments including vacuum, oxygen, hydrogen gas, and moist air. It was shown that tensile ductility and fatigue crack growth behavior depend on composition, type and degree of long range order, environment, humidity level, and frequency. All cases of embrittlement were ultimately traceable to the interaction of H with the lattice.

A recent overview of the hydrogen embrittlement of iron aluminides can also be found in Ref. [72]. Those authors employed a brand new method of in-situ nanoindentation involving hydrogen charging to study the Fe${}_{3}$Al intermetallics with a D0${}_{3}$ structure. They found the influence of hydrogen behind the reduction of Young’s moduli of the studied alloys in agreement with the hydrogen-enhanced decohesion (HEDE) model. They also reported results of measurements of the pop-in load which indicate a drastic decrease after cathodic charging in samples with low Cr content. Chromium is, therefore, one of the elements that are studied as possible ternary additions, reducing the negative impact of hydrogen. In particular, surface- and bulk-related effects were investigated by the same nanoindentation technique in Cr-containing samples of Fe${}_{3}$Al in Ref. [73]. The authors focused on different characteristics including the Young’s modulus, Gibbs-free energy for homogeneous dislocation nucleation (HDN) and velocity of dislocations. The results showed not only the reduction of Young’s moduli of the studied alloys due to the hydrogen (HEDE), but indicate also a decrease of the energy needed for HDN in the dislocation-free samples due to the presence of Cr.

Recently, there have been a few other studies aimed at examining the actual mechanism of hydrogen-caused embrittlement in Fe-Al-based materials. As far as theoretical research is concerned, the energetic stability, electronic structure, and magnetism of the *M*Al, $M_{3}$Al alloy (*M* = Fe, Ni) and variant surfaces of *M*Al (001) with and without hydrogen atoms were investigated by quantum-mechanical calculations in Ref. [74]. The hydrogen atoms were found more energetically stable in the bridge and octahedral site in *M*Al and $M_{3}$Al alloys, respectively. But it should be noted that A. A. Mubarak in Ref. [74] performed the calculations for Fe${}_{3}$Al in the L1${}_{2}$ structure (the ground-state structure of Ni${}_{3}$Al) and not in the D0${}_{3}$ structure in which Fe${}_{3}$Al crystallizes. The absorption of hydrogen atoms was found expanded resulting in embrittling of the studied host alloys. As another consequence of the absorption of H atoms, the authors reported changes in the interlayer spacing in studied surfaces and lower local density of states and magnetic moments in the surface and subsurface layers. It is also worth mentioning that the calculated concentration of hydrogen, 20 at.% in the case Fe${}_{3}$Al is so high that the results are more relevant for hydrides than a lower/dilute concentration of hydrogen.

Another first-principles study focused on thermodynamic stability and thermal properties of Fe${}_{3}$Al, Fe${}_{3}$AlC, and hypothetical Fe${}_{3}$AlX (X = H, B, N, O) compounds was published in Ref. [75]. Leaving aside the fact that the studied H concentration was also very high (20 at.%), the authors did not study H-loaded Fe${}_{3}$Al in the D0${}_{3}$ structure in which Fe${}_{3}$Al crystallizes under ambient conditions. The properties of Fe${}_{3}$AlH were computed for a κ-carbide Fe${}_{3}$AlC structure (E2${}_{1}$) which is an L1${}_{2}$ binary 3:1 fcc-based structure with a H interstitial in a body-centered octahedrally coordinated position. It is, nevertheless, noteworthy that the authors highlighted a positive role of carbon when suppressing the H embrittlement. It is known that the carbon containing dual-phase iron aluminides exhibit a relative improvement in high temperature strength as well as a reduction in the probability of hydrogen embrittlement (HE) over the single-phase iron aluminides (see, e.g., an assessment by Rao [76]).

Regarding experimental research, an effect of composition on hydrogen permeation in Fe–Al alloys was studied in Ref. [77] by comparing Fe-24wt%Al and Fe-24wt%Al-1wt%C intermetallic alloys. The electrochemical permeation technique was used to evaluate hydrogen permeability, apparent diffusivity, and solubility. An increase in carbon and aluminum contents resulted in a decrease of hydrogen permeability. Furthermore, the improved machinability of C-containing alloys was suggested to be related to a reduced susceptibility to hydrogen embrittlement in the presence of carbon. Next, the results of an in-situ microscale examination of hydrogen effect on fracture toughness in B2- and D0${}_{3}$-ordered iron aluminides intermetallic alloys were reported in Ref. [78]. The H-embrittlement in Fe${}_{3}$Al and FeAl was investigated by microcantilevers bending tests with the cantilevers loaded in-situ in an environmental scanning electron microscope either with water vapor to promote hydrogen uptake and/or with a high vacuum. For both materials, the hydrogen is found to reduce the maximum bearing load and enhance the cracking process. The in-situ electrochemical micropillar compression and nanoindentation techniques were used also when studying hydrogen embrittlement in FeAl in Ref. [79]. A reduction in the pop-in load was shown for both experiments due to in situ hydrogen charging. Clear evidence is provided that H atoms facilitate homogeneous dislocation nucleation.

Our paper aims at examining the H-related room-temperature embrittlement in the stoichiometric Fe${}_{3}$Al phase with the D0${}_{3}$ structure employing quantum-mechanical molecular dynamics. Our choice of the ab initio methodology was motivated by the fact that other computational approaches, such as those based on interatomic potentials, require suitable potentials which are difficult to obtain for our magnetic Fe-Al-H ternary system with interstitials and a mixed bond type. Furthermore, a majority of previous theoretical calculations (see some of them mentioned above) were focused on different Fe-Al materials, i.e., not Fe${}_{3}$Al with the D0${}_{3}$ structure, and were mostly performed for static lattices without thermal vibrations and/or without external straining up to the point of fracture.

## 2. Methods

Our first-principles molecular-dynamics (FP-MD) calculations were performed employing quantum-mechanical Vienna ab-initio simulation package (VASP) [80,81], which is based on the density functional theory (DFT) [82,83]. The exchange correlation energy was approximated by the generalized gradient approximation (GGA) with parametrization of Perdew and Wang, PW-91 [84], in combination with the Vosko–Wilk–Nusair correction [85] and the projector-augmented waves (PAW) potentials [86]. This specific set-up was chosen as it correctly predicts the ground state of Fe${}_{3}$Al to be the D0${}_{3}$ structure (its energy is lower than that of Fe${}_{3}$Al with the L1${}_{2}$ structure by about 5.5 meV/atom [87]). When integrating over the Brillouin zone, the Methfessel–Paxton method [88] of the first order was adopted with a smearing width 0.1 eV. The electronic loops were considered as self-consistent when the energy difference between two consequent steps was below 10${}^{-5}$ eV. The time step for the FP-MD simulations was set to 2 fs.

**Figure 1:**
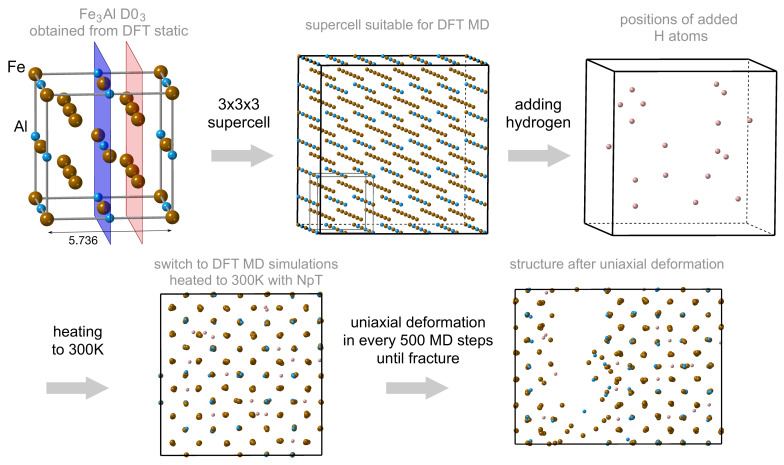
A schematic visualization of our ab initio molecular-dynamics computational set-up aimed at determing the impact of H atoms on Fe${}_{3}$Al under uniaxial deformation along the 〈001〉 crystallographic direction.

Our first-principles molecular-dynamics simulations were performed for supercells that were based on a 3 × 3 × 3 multiple of the fully-optimized 16-atom cube-shape cell of Fe${}_{3}$Al shown in Figure 1, i.e., 432 atoms of Fe and Al (in the case of Fe${}_{3}$Al without interstitial H atoms). For such large supercells we used the plane-wave basis set expanded up to either 250 eV or 350 eV (the cut-off energy) and only a single k-point (the Γ point) and we employed the VASP version pre-compiled for the Γ-point only. The higher value of the cut-off energy was employed during the equilibration phase employing the barostat. This choice was motivated by the fact that the barostat uses the stress tensor components as computed by the VASP code and reliable stress values require higher cut-off energies. During the equilibration we used the NpT algorithm (MDALGO = 3 and ISIF = 3 the VASP computational parameters). The NpT was included only during the heating and equilibration process and not for the tensile-test simulations. When simulating tensile loading, we have not used stress values provided by the VASP code as components of the tress tensor but we have evaluated the stresses from the energy-strain dependences, instead. As the stress tensor was not used in the tensile test simulations, a lower value of cut-off energy equal to 250 eV was sufficient for obtaining reliable results. The same computational set-up was applied to supercells with hydrogen, in particular one H atom (1/433 = 0.2309 at.% of H), two H atoms (0.4608 at.%), six H atoms (1.3699 at.%), 12 H atoms (2.7027 at.%), and 18 H atoms (4.0000 at.%). The hydrogen atoms were originally randomly inserted into octahedral sites inside the supercells. The spin polarized calculations were used for all present simulations and all simulations were initially started in a ferromagnetic state. Our choice of octahedral interstitial sites was motivated by very recent theoretical results of Mubarak [74] who obtained the octahedral sites energetically preferred over the tetrahedral ones in Fe${}_{3}$Al (albeit in the L1${}_{2}$ phase and for the H concentration of 20 at.%). Here it is worth mentioning that a critical hydrogen concentration for embrittling Fe${}_{3}$Al was estimated from experiments in Ref. [89] to be about 0.002 at.%. Our lowest simulated hydrogen concentration (0.2309 at.%) is admittedly two orders of magnitude higher than that estimated in Ref. [89] but is should be mentioned that our simulated processes are essentially elastic deformations (without any plasticity processes active) in a bulk (no grain boundaries) while the very low critical concentration of 0.002 at.% is likely related to processes involving plasticity and often also grain boundaries.

The obtained supercells (with and without hydrogen) were heated to temperature 300 K within 6000 steps (the heating process) and kept at a constant temperature for another 6000 steps to find the structure equilibrium state for the given temperature (the constant temperature process). A higher cut-off energy of 350 eV was used at this stage because the barostat was used and the evaluation of the stress-tensor components by the VASP code requires higher cut-off energies.

The stress-strain response was obtained under the uniaxial deformation (UD) method along the direction 〈001〉 when the supercell is deformed (elongated) along the selected direction while the other two perpendicular supercell dimensions remain constant (equal to the strain-free fully relaxed value). A lower cut-off energy value of 250 eV was used for these simulations of tensile deformations. The details about the UD deformation model are described in the work of Černý et al. [90]. This loading type usually leads to a triaxial loading state [90]. This is particularly true for the Fe${}_{3}$Al with the D0${}_{3}$ structure when our DFT static-lattice simulations of the uniaxial deformation resulted in a hydrostatic loading conditions as described in our previous work [42]. The tensile strength was determined for each atomic configuration as the maximum at the stress-strain dependence and it is marked as $\sigma_{\mathrm{ts}}^{\mathrm{UD}}$.

All simulations of deformations were performed under a temperature of 300 K and we also fixed the lateral dimensions to prevent oscillations of the external pressure. The deformations were realized via incremental increases of the lattice parameter $a_{z}$ with the step of 0.01 Å where each individual step consists of 500 FP-MD steps. We performed 35–40 deformation steps to obtain a stress-strain curve, i.e., about 200,000 of the FP-MD steps for each studied concentration of H (0, 1, 2, 6, 12, and 18 H atoms).

We note that there are other computational models for simulating the initiation of fracture (see, e.g, Refs. [91,92,93,94,95,96]), such as those based on rigid grain shifts (RGS) [97,98,99], uniaxial loading (when the Poisson contraction is included) [100], or different types of atomic relaxations [101] and involvement of the surrounding matrix [102]. In our methodology the simulated system chooses the fracture plane itself (unlike in the RGS) and we did not permit a lateral (Poisson’s) contraction (as in the uniaxial loading model) because we expect the surrounding matrix to influence the lateral sizes. We note that all the above-mentioned models are only approximates and the real response would be in between values determined via these methods.

## 3. Results

### 3.1. Hydrogen Position in the Fe${}_{3}$Al with the D0${}_{3}$ Structure

**Figure 2:**
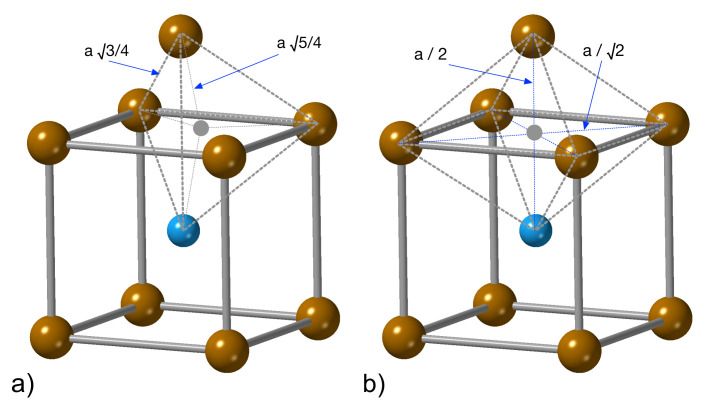
A schematic visualization of selected tetrahedral (**a**) and octahedral (**b**) interstitial sites (shaded small circles) in the D0${}_{3}$ structure of Fe${}_{3}$Al.

In the first stage we investigated the hydrogen positions in the D0${}_{3}$ structure. According to the crystal symmetry and the already published data, there are two types of positions of hydrogen atoms, namely the octahedral and tetrahedral site. Examples of both types of positions are depicted in Figure 2. According to the previously published results obtained from the static DFT calculations of Fe${}_{3}$Al with the L1${}_{2}$ structure and 20 at.% of hydrogen atoms [74], the H atoms prefer the octahedral sites. Therefore, we initiated our MD runs with the H atoms in these positions but it should be emphasized that we computed the properties of Fe${}_{3}$Al in its ground-state D0${}_{3}$ structure. We evaluated all hydrogen positions in the last 1000 FP-MD steps at a constant temperature of 300 K for the hydrogen concentration of 0.23% (only one hydrogen atom in the simulation supercell). During this simulation, we monitored the distances between the hydrogen atom and its neighboring atoms (Fe or Al) and compared them with the interatomic hydrogen-Fe(Al) distances in different interstitial positions defined in a static lattice.

**Figure 3:**
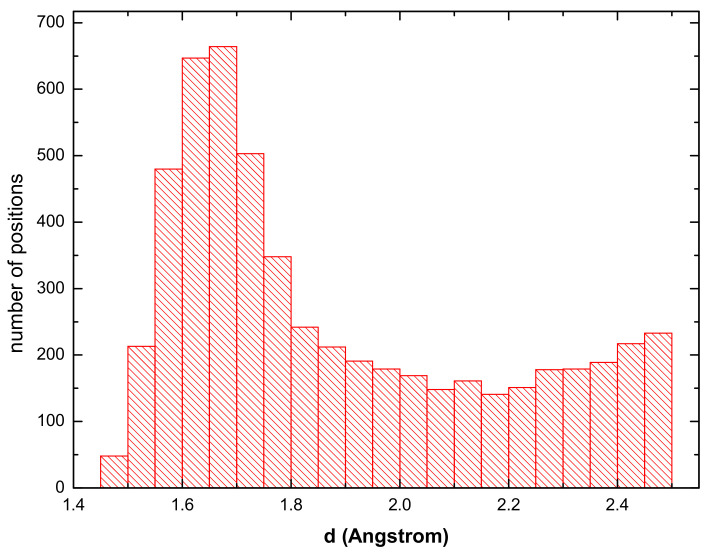
The distances between the H atoms and the neighbors from our MD run.

The H atom in a tetrahedral site has four neighboring atoms of Fe(Al) located at the distance $d_{4}^{\mathrm{tet}}$ = $\sqrt{5}/4$*a* while the octahedral site has six atoms in the close distance when four of them are located at the distance *d*${}_{4}^{\mathrm{oct}}$ = *a*/$\sqrt{2}$, and two at *d*${}_{2}^{\mathrm{oct}}$ = *a*/2. Considering the minimum-energy lattice parameter for the supercell with the hydrogen concentration of 0.23% (17.04 Å) divided by six (in order to obtain the lattice parameter *a* as in Figure 2), we get the following values of the distances between the H interstitial and the neighboring atoms: *d*${}_{4}^{\mathrm{tet}}$ = 1.59 Å, *d*${}_{4}^{\mathrm{oct}}$ = 2.01 Å and *d*${}_{2}^{\mathrm{oct}}$ = 1.42 Å. The interatomic distances obtained during the 1000 FP-MD steps at the constant temperature of 300 K for the hydrogen concentration of 0.23% are shown in Figure 3. The most frequently occurring distances between the hydrogen and the neighboring atoms are within the interval *d*∈〈1.6;1.7〉 Å which matches very well the expected value for the tetrahedral site (*d*${}_{4}^{\mathrm{tet}}$ = 1.59 Å). Figure 3 does not contain any other maxima and there is no evidence of the distance close to 1.42 Å which would indicate H in the octahedral sites. On the basis of these results we assume that the H atoms prefer the tetrahedral sites.

Our finite-temperature (T = 300 K) quantum-mechanical molecular-dynamics calculations for 0.23 at.% of hydrogen in Fe${}_{3}$Al with the D0${}_{3}$ structure show that the hydrogen atoms strongly prefer the tetrahedral sites. Our findings disagree with the H preference for octahedral sites reported by Mubarak [74] but the ab initio calculations in Ref. [74] were performed for Fe${}_{3}$Al with the L1${}_{2}$ structure, i.e., not the ground-state D0${}_{3}$ structure of Fe${}_{3}$Al. Our results are, on the other hand, in agreement with the findings of Fu and Painter [103] obtained for hydrogen atoms in the stoichiometric FeAl with the B2 structure (tetrahedral sites are preferred). Our findings also agree with the fact that the diffusion of H in the elemental Fe proceeds via tetrahedral sites [104]. The complexity of the thermodynamic preference of hydrogen atoms with respect to different types of interstitial positions manifests itself also in the results of Johnson and Carter [105]. They found a complex behavior of hydrogen atoms which differs for H atoms (i) absorbed at the surface of FeAl, (ii) diffused into the subsurface atomic planes, or (iii) diffusing deeper into the bulk FeAl. In the case of (001) surface termination, the hydrogen atoms occupy tetrahedral positions among the subsurface atomic planes while for the (110) surface termination, the H atoms diffuse into octahedral interstitial positions among the subsurface layers.

In order to shed more light on this complex issue, we located one H atom in a geometrically-ideal octahedral position and then minimized the energy with respect to atomic positions. A smaller supercell as a 2 × 2 × 2 multiple of cubic-shape 16-atom cell of Fe${}_{3}$Al was used.

**Figure 4:**
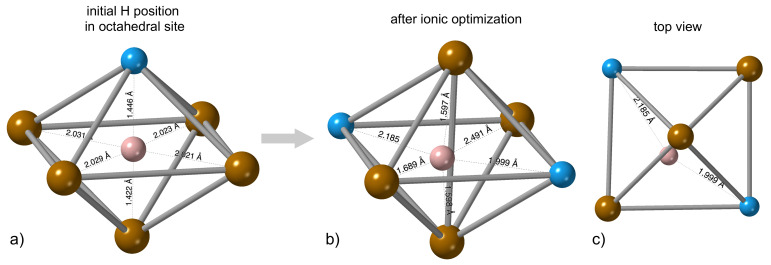
The relaxation (i.e, an energy minimization with respect to) H position from the octahedral site with one Al atom (**a**) into a neighboring octahedral environment with two Al atoms where the H atom moves away from an ideal symmetric octahedral position into a tetrahedral-like site (**b**) that is shown also from another view-angle as a top-view in sub-figure (**c**).

The H atoms moved into a neighboring octahedral environment with two Al atoms (see Figure 4a) during this structural relaxation and slipped from an octahedral interstitial position into a tetrahedral-like interstitial position (see Figure 4b,c). Therefore, the tetrahedral-like positions are preferred by the H atoms even in the case of static lattices, i.e., similarly as in molecular-dynamics simulations with thermal atomic vibrations.

**Figure 5:**
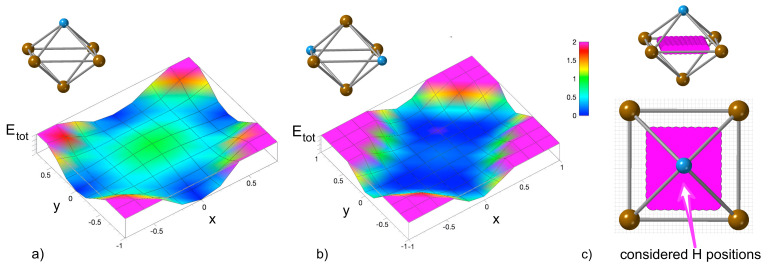
The energy surface corresponding to different locations of the H atom within a static lattice (modeled by 16-atom supercell) with the H atom inside an octahedron with only one Al atom (**a**) and within an octahedron with two Al atoms (**b**). The computed H positions are schematically visualized in part (**c**). The color-coded changes in the total energy are in eV. The *x* and *y* axes represent the shifts in the cartesian coordinates (Å) from the geometrically ideal octahedral site that is located in the origin (0,0).

In order to examine the energetics of these changes, we fixed the lattice completely, i.e., no relaxation was allowed, and the total energy was determined for one H atom within a 16-atom computational cell for a dense set of positions for both the middle plane within the octahedral environment containing either one Al atoms or with two of them. The energy surfaces are shown in Figure 5. The ideal octahedral positions represent energy maxima for the H atom in both cases with the maximum being more pronounced in the former case, see Figure 5a. The tetrahedral-like sites represent energy minima. The final position of the H atom shown in Figure 5b,c is not a typical tetrahedral position. It is located inside the tetrahedron but it is very close to the octahedral site. It is worth noting that when we analyzed the inter-atomic distances (see them in Figure 4) we found that three of the distances from the H to its neighbors are within the range of 〈1.6, 1.7〉 Å.

The other atoms exhibit interatomic distances equal or above 2.0 Å and cover a wider range of values. This means that the most frequently observed distance in the statistics obtained from the FP-MD simulations should be close to a value of 1.6 Å. This trend is visible in the histogram of distances in Figure 3 where the most frequently found distance is 1.65 Å. Thus, the static DFT simulations and the FP-MD data are fully consistent.

### 3.2. Effect of Hydrogen on the Stress-Strain Behavior

In this subsection we analyze the effect of hydrogen on materials characteristics of Fe${}_{3}$Al as obtained from molecular dynamics simulations at T = 300 K. We start with discussing the averaged volume of our computational supercells $\langle V_{\mathrm{eq}}\rangle$ obtained for the equilibrated stress-free states for different H concentrations. The computed values are summarized in Table 1. They show that the lattice expands with the rate equal to about 9.3 Å${}^{3}$ per 1 at.% H. The computed lattice expansion for higher hydrogen concentrations is in qualitative agreement with a previous theoretical study by Fu and Painter [103] which showed a yet bigger lattice expansion for significantly higher hydrogen concentrations (20 at.%). In contrast to this trend, the total magnetic moment per supercell decreases for increasing H concentrations with the rate equal to about −3.9 $\mu_{B}$ per 1 at.% H.

**Figure 6:**
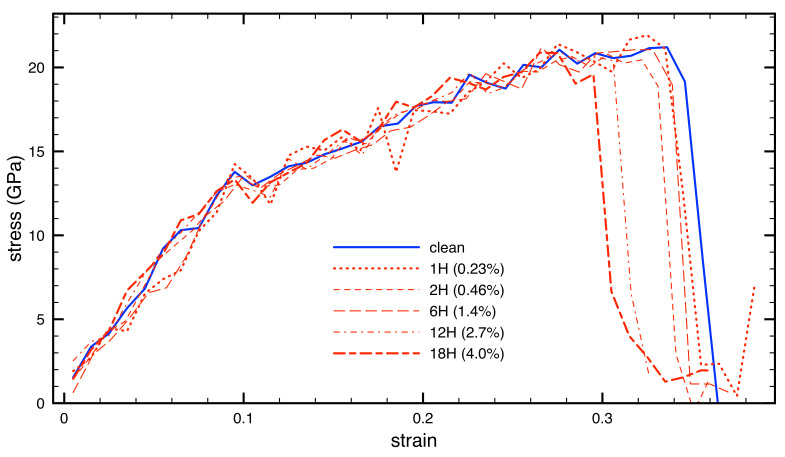
The computed stress-strain dependencies for different H concentrations.

Next we compare the stress-strain response of Fe${}_{3}$Al with and without hydrogen. The molecular-dynamics results are presented in Figure 6 where all stress-strain data are summarized. The trends reveal a decrease of the maximum strain $\epsilon_{\max}$ for higher hydrogen concentrations. The actual values are summarized in Table 1.

While the H-free Fe${}_{3}$Al has a maximum strain $\epsilon_{\max}$ equal to 0.336, it is reduced to 0.285 (reduction by 15%) in the case of the highest hydrogen concentration considered here (4 at.% of H). However there is hardly any reduction for smaller hydrogen concentration. In particular, the values for up to 0.23 at.% H are (within an expected error bar of our calculations) equal to the value computed for the H-free Fe${}_{3}$Al. The computed values of the theoretical tensile strength $\sigma_{\mathrm{ts}}^{\mathrm{UD}}$ exhibit a less clear trend with variations within quite a narrow range when compared with the maximum strains discussed above.

Last but not least, it is interesting to see that the individual stress-strain curves for different hydrogen concentrations are very similar for lower strains, nearly up to the point of fracture for the highest H concentration. However these trends reflect global characteristics of the simulated supercells and the local concentrations, e.g., close to a plane of fracture, can be significantly different and affected by diffusional thermal motion of H atoms, as well as the history of the simulated uniaxial deformation.

### 3.3. Fracture Initiation

**Figure 7:**
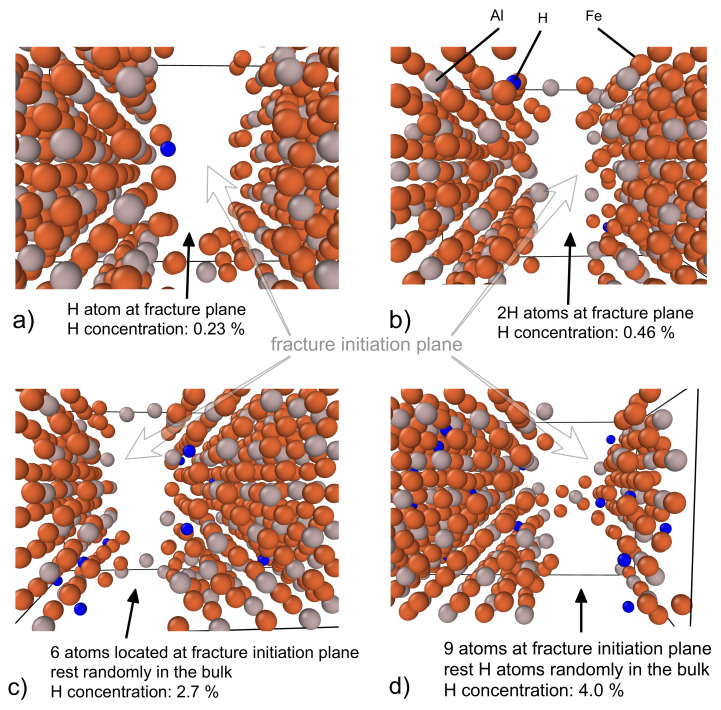
Schematic visualizations of the fracture initiation in Fe${}_{3}$Al supercells charged with a different number of hydrogen atoms. In particular, one H atoms per supercell (**a**), two H atoms (**b**), 12 H atoms (**c**), and 18 H atoms (**d**).

Unlike the static DFT simulations, the FP-MD methods are able to provide a deeper insight into the initiation of the fracture processes in the studied materials. Our analysis of fracture surfaces reveals that the hydrogen atoms are located at the fracture planes even for the lowest assumed concentration of hydrogen, see Figure 7.

The hydrogen atoms are found at the newly formed fracture surface already for the supercell containing only one H atom, see Figure 7a. For one and two H atoms all of them are at the fracture surfaces, see Figure 7a,b. As the number of H atoms grows, about one half of those that are present in the computational cell is located at the fracture surfaces, see Figure 7c,d. We interpret our findings as follows: The local fluctuation (accumulation) of hydrogen atoms contributes to the weakening of the cleavage plane during the fracture initiation. A qualitatively similar weakening of bonds between the (001) planes in the stoichiometric FeAl by interstitial hydrogen is identified in a theoretical study in Ref. [103]. As far as the hydrogen atoms at the fractured planes are concerned, when the two new surfaces are formed, the hydrogen atoms typical stay (at least temporarily) on them. Despite the fact that the fracture surfaces are not exactly planar, we can approximately call them (001) planes. These surfaces were studied by Johnson and Carter in Ref. [105] and they predict the H-Al bond length equal to 1.63 Å for 0.25 hydrogen surface coverage. This value is in an excellent agreement with our interatomic distances between the hydrogen atoms at the tetrahedral interstitial positions and Fe (or Al) atoms around (see Figure 3).

## 4. Conclusions

We performed a quantum-mechanical molecular-dynamics (MD) study of stoichiometric Fe${}_{3}$Al with the D0${}_{3}$ structure with and without hydrogen atoms under conditions of uniaxial deformation up to the point of fracture. In an attempt to elucidate a long-lasting problem of hydrogen-induced brittleness of iron-aluminides under ambient conditions, we performed our density-functional theory (DFT) MD simulations for T = 300 K. Starting with a very low hydrogen concentrations we simulated a single hydrogen atom in a supercell containing 432 atoms of Fe and Al (0.23 at.% of H) but our simulations were performed for up to 18 H atoms in the supercell (4 at.% of H). The MD calculations with the lowest concentration of H helped us to identify tetrahedral interstitial positions as being strongly preferred by the hydrogen atoms. Importantly, when combining our MD study with static-lattice simulations of interstitial site preference of H atoms, we have found that the H atom located at the octahedral site coordinated by one Al and five Fe atoms represent an energy maximum. Following the energy minimization, the H atom moves away from this interstitial site into a neighboring octahedral environment where it is surrounded by two Al and four Fe atoms. The corresponding geometrically ideal octahedral position is unstable, too, representing again an energy maximum. Therefore, the H atom eventually slips into a tetrahedral-like position with four nearest neighbors where the energy is minimized.

When simulating uniaxial deformation along the 〈001〉 crystallographic direction up to the point of fracture, we revealed that the hydrogen atoms are located at newly formed surfaces of fracture planes even for the lowest computed concentration of hydrogen. We thus show that, in the case of an elastic type of deformation, the hydrogen-related fracture initiation in Fe_3_Al is an intrinsic property which is active even if all other plasticity mechanism are absent. Importantly, the newly created fracture surfaces are partly non-planar (not atomically flat) due to thermal motion and, in particular, the H atoms create locally different environments. We are convinced that our results shed a new light on the hydrogen-induced brittleness of iron-aluminides under ambient conditions as one of the major problems associated with these otherwise very prospective materials.

## Figures and Tables

**Table 1:** The computed characteristics of Fe${}_{3}$Al with and without hydrogen atoms (with different hydrogen concentrations): An averaged supercell volume $\langle V_{\mathrm{eq}}\rangle$ for a minimum-energy stress-free state (432 Fe and Al atoms plus 0–18 H atoms), the theoretical tensile strength $\sigma_{\mathrm{ts}}^{\mathrm{UD}}$ (GPa), the corresponding maximum strain $\epsilon_{\max}$, and the total magnetic moment per the supercell (in $\mu_{B}$).

H (at.%)	0.0000	0.2309	0.4608	1.3699	2.7027	4.0000
$\langle V_{\mathrm{eq}}\rangle$ (Å${}^{3}$)	5190.91	5193.05	5195.04	5203.40	5215.15	5228.55
$\sigma_{\mathrm{ts}}^{\mathrm{UD}}$ (GPa)	21.2	21.9	20.8	21.1	20.6	20.9
$\epsilon_{\max}^{\mathrm{UD}}$	0.336	0.336	0.322	0.328	0.306	0.285
$\mu_{B}$ ($\mu_{B}$)	683.7	683.0	681.7	678.5	673.2	668.0

## Data Availability

The data presented in this study are available on request from the corresponding author.
